# Changes in the microbiological diagnosis and epidemiology of cutaneous leishmaniasis in real-time PCR era: A six-year experience in a referral center in Barcelona

**DOI:** 10.1371/journal.pntd.0009884

**Published:** 2021-11-10

**Authors:** Aroa Silgado, Mayuli Armas, Adrián Sánchez-Montalvá, Lidia Goterris, Maria Ubals, Jordi Temprana-Salvador, Gloria Aparicio, Carmen Chicharro, Núria Serre-Delcor, Berta Ferrer, Israel Molina, Vicenç García-Patos, Tomas Pumarola, Elena Sulleiro

**Affiliations:** 1 Department of Microbiology, Vall d’Hebron University Hospital, Universitat Autònoma de Barcelona, PROSICS Barcelona, Barcelona, Spain; 2 Department of Infectious Diseases-Drassanes, Vall d’Hebron University Hospital, Universitat Autònoma de Barcelona, PROSICS Barcelona, Barcelona, Spain; 3 Department of Dermatology, Vall d’Hebron University Hospital, Universitat Autònoma de Barcelona, Barcelona, Spain; 4 Department of Pathology, Vall d’Hebron University Hospital, Universitat Autònoma de Barcelona, Barcelona, Spain; 5 National Center of Microbiology, Majadahonda, Spain; Liverpool School of Tropical Medicine, UNITED KINGDOM

## Abstract

**Background:**

Leishmaniasis is a neglected disease caused by different species of the protozoa *Leishmania spp*. Cutaneous lesions are the most common clinical manifestation. This disease is prevalent in tropical and subtropical areas, including the Mediterranean basin. In Spain, *Leishmania (L*.*) infantum* is the only endemic species, but imported cases are often diagnosed. Different classical parasitological methods can be performed for cutaneous leishmaniasis (CL) diagnosis; but currently molecular techniques serve as a relevant tool for the detection and characterization of *Leishmania* parasites. We aimed to evaluate clinical and epidemiological characteristics of CL diagnosed patients by real-time PCR in a tertiary hospital over a six-year period.

**Methodology/Principal findings:**

Clinical, epidemiological and microbiological data were retrospectively collected and analyzed. In our study, CL was confirmed in 59 (31.4%) out of 188 patients by real-time PCR, showing an increase over recent years: 11 cases of CL between 2014 and 2016 and 48 between 2017 and 2019. Real-time PCR was performed on skin swabs and/or biopsies samples, with a positivity of 38.5% and 26.5%, respectively. Results were 100% concordant when biopsy and skin swab were performed simultaneously. *L*. *(L*.*) infantum* was the most frequent species detected (50%), followed by *L*. *(L*.*) major* (45%) and *Viannia* subgenus (5%), which were detected only in imported cases. *L*. *(L*.*) major* was almost entirely detected in travelers/migrants from Morocco. Multiple and atypical skin lesions were more common in imported cases than in autochthonous cases (44.4% vs. 21.8%).

**Conclusions/Significance:**

An increase in both autochthonous and imported CL cases has been observed in past years in our hospital. Molecular techniques assist in improving CL diagnosis and characterization of the *Leishmania* species, mainly in imported cases.

## Author summary

Leishmaniasis is a neglected disease caused by different species of the genus *Leishmania*. This disease is prevalent in tropical and subtropical areas, including the Mediterranean basin. In Spain, the only endemic species is *L*. *(L) infantum*, although there are cases of imported leishmaniasis due to travel and migration of people from other endemic areas. Cutaneous lesions are the most common clinical form, but *L*. *(L) infantum* can also cause mucosal or visceral involvement. Although microscopic diagnosis and skin biopsy specimens are still used in routine diagnosis for cutaneous leishmaniasis, they are time-consuming and invasive, respectively. Currently, molecular techniques, such as real-time PCR, are very sensitive tools for the detection and identification of *Leishmania* parasites. In addition, the skin swab sample seems to be a non-invasive alternative for the diagnosis of cutaneous leishmaniasis. In the present work, we describe the clinical characteristics of 59 patients diagnosed by real-time PCR of cutaneous leishmaniasis, as well as the molecular epidemiology of the causative species. We show that there has been an increase in cutaneous leishmaniasis cases, both autochthonous and imported cases, over the study period. The presence of *Leishmania* species other than *L*. *(L) infantum* is also highlighted.

## Introduction

Leishmaniases are neglected infectious diseases caused by at least 20 different species of the protozoan *Leishmania spp*. transmitted by the bite of a phlebotomine sandfly [1]. Leishmaniasis is endemic in more than 98 countries, including tropical and subtropical areas and the Mediterranean basin. More than one billion people are at risk of acquiring the infection and approximately 0.7–1 million cutaneous leishmaniasis (CL) cases are reported annually [2,3].

*Leishmania* species have a different geographic distribution around the world. Old World leishmaniasis, usually manifested as mild cutaneous disease, occurs in Africa, Asia and Europe and the main causative species are *L*. *(L*.*) tropica*, *L*. *(L*.*) major*, and *L*. *(L*.*) infantum*. New World leishmaniasis, with lesions range from mild cutaneous disease to severe mucosal lesions, occurs in America and is mostly caused by *L*. *(V*.*) braziliensis*, *L*. *(V*.*) guyanensis*, *L*. *(V*.*) panamensis*, *L*. *(L*.*) mexicana*, and *L*. *(L*.*) amazonensis* [4].

Depending on the characteristics of the parasite and the host response, various clinical forms of leishmaniases are present, such as CL, mucocutaneous leishmaniasis, visceral leishmaniasis, and post-kala-azar dermal leishmaniasis.

CL is the most frequent clinical form worldwide [5]. Because of the *Leishmania* species involved, this clinical form presents a variety of clinical features, treatment options, and different prognosis [4].

In the Mediterranean region, including Spain, *L*. *(L*.*) infantum* is the endemic species, which causes mainly CL and, less frequently, visceral or mucosal leishmaniasis (ML) [6,7]. In this country, the two species of sand flies involved in the transmission of *L*. *(L*.*) infantum* are *Phlebotomus perniciosus* and *P*. *ariasi* [8] and the main reservoir is the dog [7]. Although autochthonous transmission occurs in Spain, imported cases caused by other *Leishmania* species are often diagnosed in migrants or travelers returning from other endemic areas [4,9]. Imported cases may present atypical clinical forms that make clinical diagnosis difficult and, consequently, have different therapeutic approaches [4]. In addition, the presence of imported species and of the sand flies involved in their transmission could represent a risk of spread of this species within Spain.

CL diagnosis depends on clinical examination of skin lesions and a compatible epidemiology. The definitive diagnosis is based on direct demonstration the parasite or its genetic material [10,11] in clinical specimens, using microscopic examination (Giemsa staining, histopathology, immunochemistry) or molecular analysis [12].

Currently, molecular techniques mainly based in PCR protocols, serve as a relevant tool for the diagnosis of leishmaniasis due to their high sensitivity, specificity and performance feasibility in a wide variety of clinical samples, including non-invasive procedures [13]. Moreover, as allow the identification of *Leishmania* species [14,15], such techniques prove useful in epidemiological surveillance, outbreak characterization and optimal treatment care [16,17]. In addition, real-time PCR allows for the use of an internal control of DNA integrity and/or PCR inhibition in multiplexed reactions [18].

The objective of this study was to describe the epidemiological and clinical features of patients with autochthonous and imported CL diagnosed by real-time PCR in skin swab and/or biopsy sample in a tertiary hospital of the Mediterranean basin over a six-year period.

## Methods

### Ethics statement

Epidemiological, clinical and laboratory data were obtained from patients’ medical records in cases diagnosed with CL. Review Board approvals were obtained from the Ethics Committee of the Vall d’Hebron Research Institute, according to the principles expressed in the Declaration of Helsinki. All data were anonymized before being analyzed.

### Study population, samples and microbiological procedures

A retrospective study of patients who presented with clinical suspicion of CL between January 2014 and December 2019 at Vall d’Hebron University Hospital (Barcelona, Spain) was performed. The clinical charts of the patients were reviewed to obtain additional information including patient age and gender, presence of immunosuppression, travels, type of lesions, and microbiological results.

For CL diagnosis in typical single ulcerative skin lesions, first skin swab sample was collected and processed for real-time PCR. If the skin swab sample obtained a negative result by real-time PCR or the clinical diagnosis was unclear, a biopsy sample was collected and analyzed for real-time PCR and, when possible, for immunohistochemistry.

**Fig 1:**
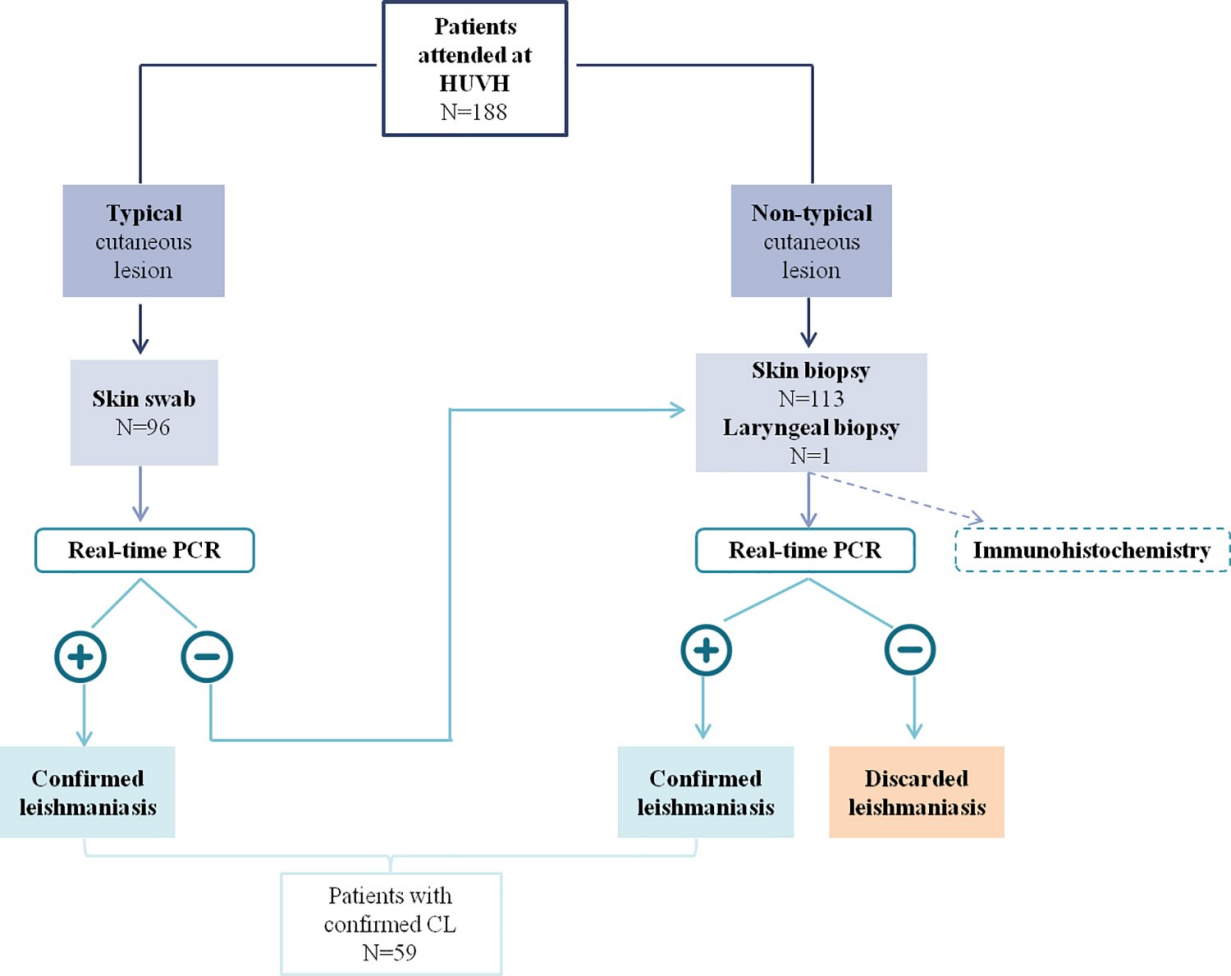
Algorithm of the samples used in this study and which assays were performed.

Definitive CL diagnosis was considered by detecting *Leishmania* DNA by real-time PCR in skin swab and/or biopsy sample (Fig 1).

### Molecular techniques

DNA extraction from skin swab was carried out via silica–membrane technology using the NucliSens easyMAG system (bioMérieux Diagnostics, Marcy-l’Etoile, France). DNA extraction from skin biopsy was performed after digestion of proteinase K, using the Qiagen Biorobot EZ1 (Qiagen, Hilden, Germany) or the MagCore Plus HF16 (RBC Bioscience, New Taipei City, Taiwan) without distinction. All protocols were performed in accordance with manufacturers’ instructions.

A duplex real-time PCR targeted to a specific region of *Leishmania spp*. kinetoplast DNA and human RNase P gene (Taq Man Human RNase P detection reagent; Applied Biosystems, Foster City, CA) was performed. Real-time PCRs were performed following the protocol described by Mary et al. [19] yet with some modifications: the final conditions in the PCR mixture were 1x Quantitec Muliplex PCR kit (Qiagen), 400 nM of both primers for *Leishmania* target, 100 nM of the TaqMan *Leishmania* probe, and 0.8x RNase P detection reagent. Reactions were performed using 5 μL of eluted DNA in a final volume of 25 μL. Amplifications were carried out in a CFX96 Touch Real-Time PCR Detection System (Bio-Rad, Hercules, CA). The cycling conditions were as follows: an initial step of 15 minutes at 95°C, followed by 40 cycles at 95°C for 15 seconds and 55°C for one minute.

In all cases, a sample was considered positive for *Leishmania* DNA when the threshold cycle (Ct) for the *Leishmania* target was <40. A sample was considered as invalid when the internal control (RNase P human gene) was inefficiently amplified. Positive and negative samples were included as external controls in each real-time PCR run.

Molecular characterization of *Leishmania* species was performed in the National Center of Microbiology laboratory (Madrid, Spain) on samples with a positive result by real-time PCR from patients who came from other countries where CL is endemic or had atypical forms of CL. Briefly, ribosomal internal transcribed spacer 1 (ITS1) was amplified and then PCR products were sequenced, following the protocol described by Kuhls et al. [20]. A dendogram was constructed in order to determine the relationship of the obtained sequence with the reference set [21].

### Immunohistochemistry

Biopsy tissues were fixed in 5% buffered formalin, embedded in paraffin blocks and cut into 4μm tissue sections. For routine diagnosis purposes, samples were stained with hematoxylin and eosin. Immunohistochemical detection of *Leishmania* amastigotes was performed on 4μm sections of formalin-fixed, paraffin-embedded tissues. The polyclonal antibody was obtained by immunizing rabbits with a *Leishmania* antigen. Briefly, sections were deparaffinized and rehydrated through graded alcohol and water. Antigen retrieval with CC1 solution (pH 8.4) for 20 minutes was performed. The sections were incubated in a humidified chamber with the antibody anti-*Leishmania* diluted 1/1000 for 32 minutes. Staining was performed with BenchMark Ultra (Ventana/Roche; Roche Diagnostics, Manheim, Germany) using the ultraVIEW Universal DAB Detection Kit (Ventana/Roche).

### Statistical analysis

Qualitative variables were expressed as absolute frequencies and percentages. Categorical variables were described as mean or median and standard deviation or interquartile range according to normal distribution. Statistical analyses were done using SPSS Statistics v22.0 (SPSS Inc, Chicago, IL).

## Results

From 2014 to 2019, a total of 210 samples from 188 patients with cutaneous lesions clinically suspected as CL were included in the study. Leishmaniasis was confirmed in 59 of 188 (31.4%) patients by real-time PCR.

Thirty-two of 59 (54%) patients were men, and the median age was 34 years (IQR 8–52.5): 20 of 59 (34%) patients were aged under 15 years; 29 (49%) patients were aged between 16 and 65 years; and the remaining 10 (17%) were aged over 65 years. Immunosuppressive conditions were detected in 11 of 59 (18.6%) patients: 10 of 11 (91%) for anti-TNF drugs and 1 patient (9%) referred alcoholism.

**Fig 2:**
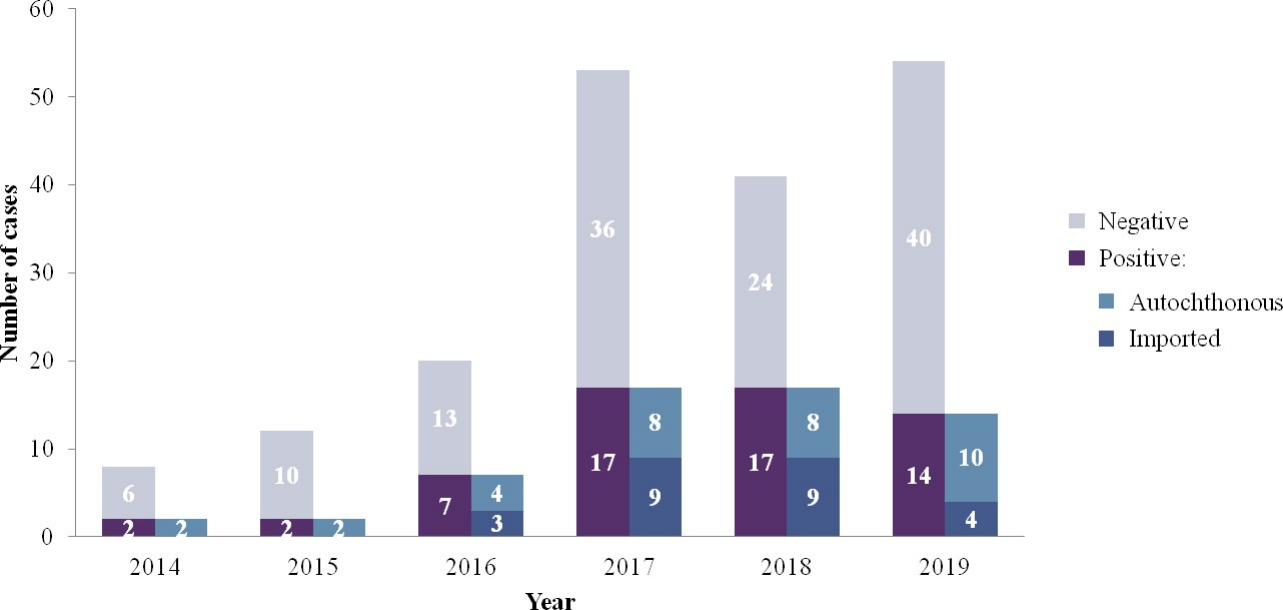
Distribution of CL cases from 2014 to 2019.

When comparing the total number of patients diagnosed in a 3-year period, 11 of 59 patients received a CL diagnosis between 2014 and 2016, and 48 of 59 patients between 2017 and 2019 (Fig 2). In the entire study period, 27 (45.8%) patients had previous travel history to a country where CL is endemic, in whom the skin lesion appears after the trip, and had a diagnosis of imported leishmaniasis. These patients reported having traveled to Morocco (20 of 27; 74%), other countries in the Mediterranean basin (4; 14.8%, including Italia, France, and Israel), Senegal (1; 3.7%), Peru (1; 3.7%) and Costa Rica (1; 3.7%).

Localized cutaneous leishmaniasis (LCL) was diagnosed in 26 of 59 (44.1%) patients, being papule the most common skin manifestation (11 of 26 patients; 42.3%). Nineteen of 59 (32.2%) patients presented with more than two lesions (multifocal leishmaniasis), of which 5 patients (26.3%) presented two different types of skin lesions: nodules and plaques (2 of 5; 40%), papules and nodules (2; 40%) or plaques and papules (1; 20%). Three of 59 (5%) patients had oral involvement (none of them with immunosuppressive status) and were considered to have ML. Clinical presentations of skin lesions, differentiating between autochthonous and imported cases, are shown in Table 1.

**Table 1:** Different clinical presentation of CL per the source of infection.

	Autochthonous (N = 32)	Imported (N = 27)	Total (N = 59)
**Local cutaneous lesion (LCL)**	**18**	**8**	**26**
Nodule	5	1	6
Papule	9	2	11
Plaque	3	4	7
Non-defined^a^	1	1	2
**Multifocal leishmaniasis**	**7**	**12**	**19**
Nodule	1	2	3
Papule	1	1	2
Plaque	1	4	5
Combination^b^	2	3	5
Non-defined	2	2	4
**Mucosal leishmaniasis**	**1**	**2**	**3**
**Non-defined** ^**a**^	**6**	**5**	**11**

^a^no data available in the patient’s medical record

^b^combination of two different types of skin lesions

Skin samples from patients with CL suspicion were distributed as follows: 113 of 210 (53.8%) were skin biopsies; 96 (45.7%) were ulcer swabs; and 1 (0.5%) was a laryngeal biopsy. *Leishmania* DNA was detected by real-time PCR in 30 of 113 (26.5%) skin biopsies and 37 of 96 (38.5%) swab samples. In 13 patients, both swab and biopsy samples were performed simultaneously, obtaining concordant results; seven (53.8%) of these patients had a positive real-time PCR result. The laryngeal biopsy also tested positive for *Leishmania* DNA.

Retrospectively, in 39 skin biopsies immunohistochemistry and real-time PCR have been performed in parallel. A total of 17 of 39 (43.6%) samples were positive with both techniques, 18 of 39 (46.1%) samples were negative by both techniques, and 4 of 39 (10.3%) samples were negative by immunohistochemistry but positive by real-time PCR.

**Fig 3:**
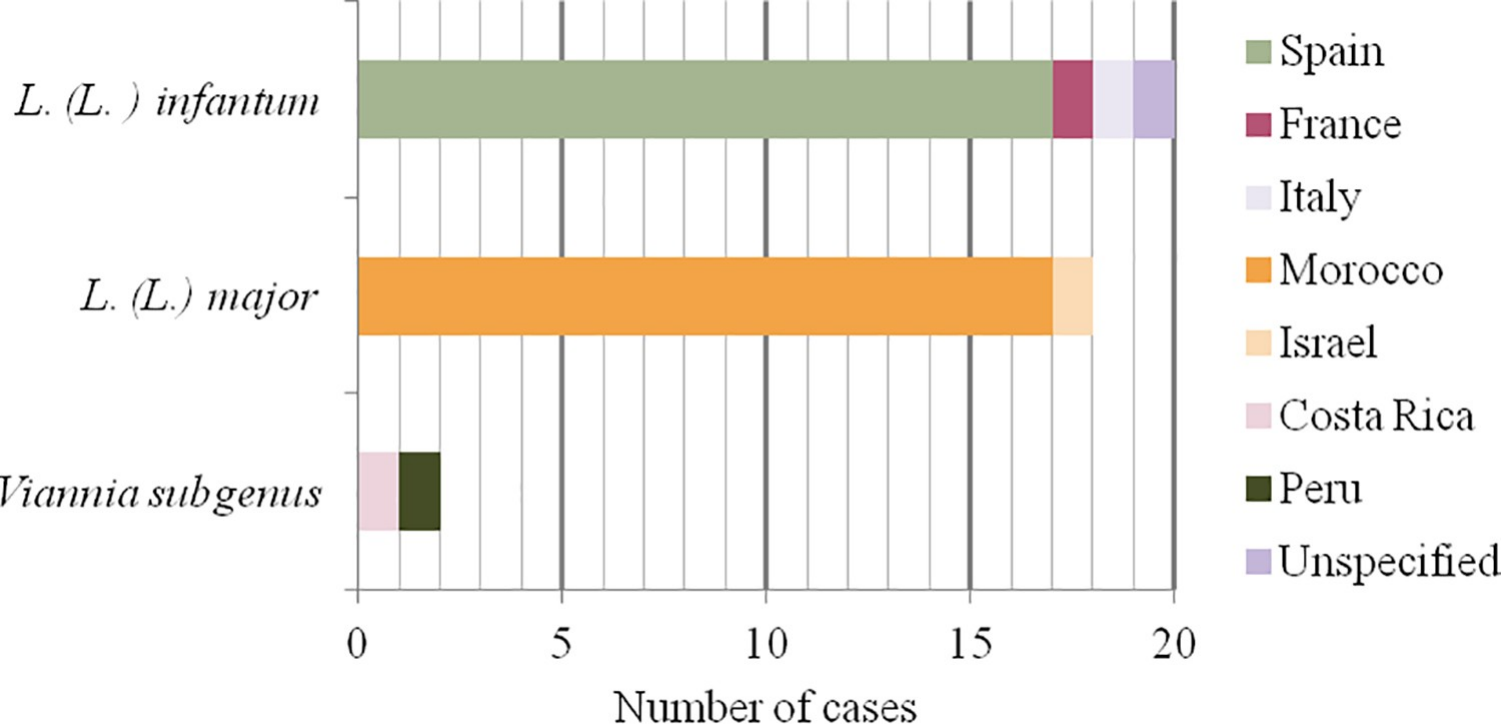
Geographical origins of different *Leishmania* species.

Species identification could not be performed in 8 of 59 (13.6%) cases and in 11 of 59 (18.6%) patients it was not performed because they were autochthonous cases with typical lesion. Molecular characterization of *Leishmania* species was determined in 40 of 59 (67.8%) confirmed patients. *L*. *(L*.*) infantum* and *L*. *(L*.*) major* were found to be the most frequent species in 50% (20/40) and 45% (18/40) of cases, respectively. Almost all the samples typed as *L*. *(L*.*) major* (17 of 18; 94.4%) were from patients in Morocco, except one sample from a patient who traveled to Israel (1 of 18; 5.6%). The vast majority of samples typed as *L*. *(L*.*) infantum* (17 of 20; 85%) were from autochthonous cases and the remaining three (3 of 20; 15%) were from patients who traveled to other Mediterranean countries. Two (5%) samples were classified under the *Viannia* subgenus, of which one corresponded with a traveller returning from Costa Rica and the other to a Peruvian migrant. The first case could be typed as *L*. *(V*.*) brazilensis* but in the second case the specific species could not be determined. The *Leishmania* species and their geographical origins are shown in Fig 3.

Clinical infections caused by *L*. *(L*.*) infantum* were mostly (10 of 20; 50%) classified as LCL (Table 2). This species was also the causative agent of multifocal CL in 4 of 20 (20%) cases and ML in 3 of 20 (15%) cases.

**Table 2:** Clinical presentation of CL according to the species identified.

	Localized CL	Multifocal CL	Mucosal CL	Non-defined^b^
***L*. *(L*.*) infantum***	10	4	3	3
***L*. *(L*.*) major***	6	7		5
***Viannia* subgenus**		2		
**Undetermined** ^**a**^	10	6		3

^a^molecular typing could not be performed

^b^no data available

*L*. *(L*.*) major* caused a LCL in 6 of 18 (33%) cases and multifocal CL in 7 of 18 (38.9%) cases. The two (100%) patients infected by *Leishmania* species from the *Viannia* subgenus were classified as multifocal CL.

## Discussion

This study shows the results of 59 patients diagnosed with CL by real-time PCR in a Barcelona-based tertiary hospital over a 6-year period.

Clinical and epidemiological presentation observed in this work were similar to that in other studies conducted in Spain, in which patients with CL were mainly adult males [22–25]. During the study period, an increase in the number of both autochthonous and imported CL cases in the last, few years was observed; this rise was similar to that in other cohorts [23,26,27]. According to data published in our country, an elevated number of immunosuppressed patients [28] and migrants/travelers from highly endemic CL settings [8], could explain this rise in leishmaniasis cases. In addition, the use of sensitive molecular techniques could have contributed to confirming presumptive clinical diagnoses and, thereby, more cases of infection [25].

In this study, leishmaniasis diagnosis was performed by real-time PCR on skin biopsies and/or swab samples. Despite the high sensitivity of molecular techniques, the performance of real-time PCR obtained in this work is quite low in general (38.5% and 26.5% for skin swab and biopsy samples, respectively). This low percentage of positivity may be due to the fact that leishmaniasis presents a great diversity of clinical manifestations [29,30], which makes its diagnostic orientation difficult if not a typical cutaneous lesion is observed with a compatible epidemiology.

Skin biopsy is the most robust sample to diagnose CL. However, swab scraping confers substantial benefits regarding biopsy (less invasive and painful, easier to perform and does not make permanent scar) and should be recommended as the first approach [31,32]. Our data show a higher percentage of positivity for *Leishmania* DNA detection in swab samples than the biopsy sample (38.5% vs. 26.5%, respectively). The lower positivity of real-time PCR on skin biopsy samples may be due to the fact that this type of sample is obtained to diagnose atypical lesions that may correspond to clinical entities other than leishmaniasis. In our study, few patients had both skin biopsy and swab sample, with 100% concordance between the real-time PCR results of both samples. Yet, few attempts have been made to simultaneously compare the utility of different lesion sampling procedures for PCR-based CL diagnosis; sensitivity reached approximately 93–98% when a swab sample was used [31,33].

In 39 samples, both real-time PCR and immunohistochemistry could be performed in parallel; however, both techniques attained only 89% agreement, with 4 negatives for immunohistochemistry. This finding supports the use of real-time PCR as a diagnostic method for CL, with particular utility in disease types with low parasitic loads [34]. In a large number of studies, sensitivity results obtained from PCR protocols is better when compared with classical parasitological methods, including immunohistochemistry, with values reaching higher than 90% [15,25,35,36]. Due to high sensitivity, accessibility and simplicity, molecular assays have been included in diagnostic strategies within many settings.

In our cohort, at least four *Leishmania* species belonging to both *Leishmania* and *Viannia* subgenus caused infections in patients with CL. However, the vast majority of infections were due to species from the *Leishmania* subgenus; *L*. *(L*.*) infantum* was the most frequent causative agent (50% of cases). As expected, in all autochthonous cases whose samples could be typed, *L*. *(L*.*) infantum* was identified as the causative agent.

Species identification of imported cases, including *L*. *(L*.*) infantum*, *L*. *(L*.*) major* and *Viannia* subgenus, was fully consistent with the travel history of all patients.

In our clinical practice, molecular typing was not performed in autochthonous patients with a typical single ulcerative lesion, and these infections were interpreted as *L*. *(L*.*) infantum*. If we considered these autochthonous patients and molecular typed-confirmed cases, the percentage of *L*. *(L*.*) infantum* would rise from 50% to 60.8%, and *L*. *(L*.*) major* and *Viannia* would drop from 45% to 35.3% and from 5% to 3.9%, respectively. Similar detection rates of *Leishmania* species, in both autochthonous and imported cases, were reported in a study done by Merino-Espinosa et al. from another Spanish hospital (70% *L*. *(L*.*) infantum* vs. 30% *L*. *(L*.*) major*) [37]. To know the circulating *Leishmania* species will contribute to a better understanding of the epidemiology of leishmaniasis [21].

Most of our patients infected with *L*. *(L*.*) major* came from Morocco. In the last, few decades, an important epidemic focus of CL has occurred in the arid pre-Saharan regions. *L*. *(L*.*) major* is the most important causative agent of these outbreaks in the southern of Morocco (79% between 2015 and 2016) resulting primarily in multifocal skin lesions [38,39]. In focus, *P*. *papatasi* sand fly, the main vector of *L*. *(L*.*) major* [40], is distributed in large regions of Spain [41], making possible that this *Leishmania* specie could become established as an autochthonous specie in our country. Thus, infected travelers/migrants could also represent potential reservoir hosts for anthroponotic *Leishmania* parasite acquired abroad [42]. In this sense, appropriate surveillance network is essential in order to monitoring the potentially introduction of non-endemic *Leishmania* species in endemic areas with local autochthonous transmission [43].

Characterization of causative species of *Leishmania* is relevant when deciding adequate treatment and managing the disease [4,9,14]. CL caused by Old World *Leishmania* species tends to resolve spontaneously, but CL caused by New World species causes serious infections and only a few percentage of cases resolve spontaneously [12].

Clinical infections caused by *L*. *(L*.*) infantum* were mostly (51.4%) classified as LCL. However, there were three cases of ML, with *L*. *(L*.*) infantum* being the etiological agent. Although mucosal involvement is not common [24], cases of ML caused by *L*. *(L*.*) infantum* have been reported in Spain and other European regions [44].

Our study does, however, have limitations due to its retrospective nature. For example, the study lacks information regarding some patients because we had no clinical and/or epidemiological information. Additionally, in those patients with skin biopsy sample, not in all cases we were able to obtain information regarding the immunohistochemistry technique. Due to the low number of skin swabs and biopsies samples collected in parallel, a significant difference in real-time PCR positivity could not be determined.

## Conclusions

Autochthonous and imported cases of CL diagnosed by real-time PCR have been increasing over the last, few years.

Due to the high number of imported leishmaniasis cases detected, other *Leishmania* species than *L*. *(L*.*) infantum* was also increased, especially *L*. *(L*.*) major* in travelers and/or migrants from Morocco.

Molecular characterization provides important epidemiological information about patients from other geographical regions or with atypical forms of leishmaniasis, as well as what approaches to undertake for optimal treatment care.
